# Supplementary material for: Real-World Data on Severe Cutaneous Adverse Reactions to Drugs
Source: Pharmaceuticals (Basel). 2025 Dec 22;19(1):21. doi: 10.3390/ph19010021 (PMC12845191; doi:10.3390/ph19010021)
Supplement: Supplementary file 1 [file pharmaceuticals-19-00021-s001.zip › pharmaceuticals-4000105-supplementary.pdf]

Table S1. Annual distribution of SRs for SMQ SCAR and PTs.

PT	2019 (9 months)	2020	2021	2022	2023	2024	2025 (3 months)	Total number
SMQ - SCAR	101	440	659	1229	1869	2164	549	7011
Drug reaction with eosinophilia and systemic symptoms	5	80	107	219	359	407	95	1272
Toxic skin eruption	47	87	147	181	287	304	81	1134
Toxic epidermal necrolysis	12	47	60	116	230	266	72	803
Erythema multiforme	9	45	58	131	189	226	51	709
Stevens-Johnson syndrome	8	31	58	121	196	223	60	697
Dermatitis bullous	6	53	74	95	165	194	57	644
Acute generalized exanthematous pustulosis	2	37	50	113	118	138	28	486
Dermatitis exfoliative generalised	2	47	42	70	98	147	28	434
Cutaneous vasculitis	0	2	24	79	105	122	45	377
Skin necrosis	5	20	20	58	61	52	16	232
Dermatitis exfoliative	2	8	19	24	36	55	10	154
Exfoliative rash	2	5	9	36	29	26	10	117
Epidermal necrosis	1	7	4	6	10	18	3	49
SJS-TEN overlap	0	0	2	4	10	16	3	35
Bullous haemorrhagic dermatosis	0	2	6	8	9	8	1	34
Severe cutaneous adverse reaction	0	0	2	1	9	16	3	31
Generalized bullous fixed drug eruption	0	0	1	2	9	5	2	19
Oculomucocutaneous syndrome	0	0	2	5	3	3	0	13
Target skin lesion	0	4	1	3	3	1	1	13
Erythrodermic atopic dermatitis	0	0	0	0	1	1	0	2

Table S2. Structure of outcomes for SMQ SCAR and top-10 PTs.

**Outcome**	**SMQ - SCAR n (%) Total number —7011**	**DRESS n (%) Total number —1272)**	**TSE n (%) Total number —1134**	**TEN n (%) Total number —803**	**EM n (%) Total number —709**	**SJS n (%) Total number —697**	**DB n (%) Total number —644**	**AGEP n (%) Total number —486**	**DEG n (%) Total number — 434**	**CV n (%) Total number —377**	**SN n (%) Total number —232**
Death	407 (5.8)	54 (4.2)	4 (0.4)	225 (28.0)	8 (1.1)	83 (11.9)	7 (1.1)	6 (1.2)	6 (1.4)	7 (1.9)	9 (3.9)
Condition unchanged	653 (9.3)	79 (6.2)	104 (9.2)	87 (10.8)	60 (8.5)	69 (9.9)	66 (10.2)	40 (8.2)	41 (9.4)	41 (10.9)	40 (17.2)
Condition improved	1880 (26.8)	371 (29.2)	359 (31.7)	165 (20.5)	218 (30.7)	184 (26.4)	110 (17.1)	154 (31.7)	103 (23.7)	95 (25.2)	46 (19.8)
Recovery with consequences	91 (1.3)	6 (0.5)	5 (0.4)	20 (2.5)	11 (1.6)	14 (2.0)	7 (1.1)	6 (1.2)	8 (1.8)	4 (1.1)	10 (4.3)
Recovery without consequences	2457 (35.0)	562 (44.2)	502 (44.3)	159 (19.8)	243 (34.3)	164 (23.5)	173 (26.9)	222 (45.7)	166 (38.2)	145 (38.5)	58 (25.0)
Unknown	1523	200	160	147	169	183	281	58	110	85	69

Table S3. List of drugs revealed in all PTs.

PT	Initial number of drugs	Final total number of drugs*
Acute generalised exanthematous pustulosis	946	905
Bullous haemorrhagic dermatosis	50	47
Cutaneous vasculitis	558	549
Dermatitis bullous	909	892
Dermatitis exfoliative	188	181
Dermatitis exfoliative generalised	434	430
Drug reaction with eosinophilia and systemic symptoms	2729	2663
Epidermal necrosis	71	69
Erythema multiforme	1015	998
Erythrodermic atopic dermatitis	2	2
Exfoliative rash	145	140
Generalized bullous fixed drug eruption	32	32
Oculomucocutaneous syndrome	19	18
Severe cutaneous adverse reaction	43	42
SJS-TEN overlap	53	52
Skin necrosis	352	340
Stevens-Johnson syndrome	1164	1127
Target skin lesion	20	20
Toxic epidermal necrolysis	1670	1606
Toxic skin eruption	1698	1656

*After exclusion of drugs without ATC codes.

Table S4. ATC 1st level groups revealed in SCARs.

ATC 1st Level Group	n Total number - 11376	%
A	748	6.6
B	726	6.4
C	673	5.9
D	308	2.7
G	69	0.6
H	194	1.7
J	3442	30.3
L	2677	23.5
M	577	5.1
N	1509	13.3
P	116	1.0
R	186	1.6
S	10	0.1

V	141	1.2

Table S5. Distribution of ATC 1st level groups among PTs (total number - 11376).

PT	ATC 1 Level Group													
	**A** n (%)	**B** n (%)	**C** n (%)	**D** n (%)	**G** n (%)	**H** n (%)	**J** n (%)	**L** n (%)	**M** n (%)	**N** n (%)	**P** n (%)	**R** n (%)	**S** n (%)	**V** n (%)
Acute generalized exanthematous pustulosis	40 (4.4)	91 (10.1)	61 (6.7)	36 (4.0)	10 (1.1)	27 (3.0)	338 (37.4)	97 (10.7)	47 (5.2)	90 (9.9)	23 (2.5)	19 (2.1)	0	26 (2.9)
Bullous haemorrhagic dermatosis	0	20 (42.6)	2 (4.3)	0	0	0	6 (12.8)	9 (19.2)	3 (6.4)	7 (15.0)	0	0	0	0
Cutaneous vasculitis	21 (3.8)	70 (12.8)	58 (10.6)	21 (3.8)	7 (1.3)	13 (2.4)	107 (19.5)	187 (34.1)	30 (5.5)	18 (3.3)	4 (0.7)	9 (1.6)	1 (0.2)	3 (0.6)
Dermatitis bullous	261 (29.3)	62 (7.0)	43 (4.8)	12 (1.4)	3 (0.3)	17 (1.9)	164 (18.4)	197 (22.1)	36 (4.0)	69 (7.7)	9 (1.0)	12 (1.4)	0	7 (0.8)
Dermatitis exfoliative	17 (9.4)	12 (6.6)	8 (4.4)	14 (7.7)	5 (2.8)	4 (2.2)	39 (21.6)	44 (24.3)	7 (3.9)	28 (15.5)	0	2 (1.1)	0	1 (0.6)
Dermatitis exfoliative generalised	20 (4.7)	26 (6.1)	22 (5.1)	87 (20.2)	5 (1.2)	5 (1.2)	76 (17.7)	135 (31.4)	13 (3.0)	24 (5.6)	3 (0.7)	8 (1.9)	0	6 (1.4)
Drug reaction with eosinophilia and systemic symptoms	124 (4.7)	136 (5.1)	188 (7.1)	14 (0.5)	11 (0.4)	35 (1.3)	1140 (42.8)	319 (12.0)	127 (4.8)	477 (17.9)	24 (0.9)	24 (0.9)	1 (0.04)	43 (1.6)
Epidermal necrosis	2 (2.9)	4 (5.8)	5 (7.3)	8 (11.6)	0	4 (5.8)	12 (17.4)	24 (34.8)	3 (4.4)	1 (1.4)	5 (7.3)	1	0	0
Erythema multiforme	36 (3.6)	56 (5.6)	43 (4.3)	62 (6.2)	9 (0.9)	16 (1.6)	230 (23.1)	357 (35.8)	61 (6.1)	90 (9.0)	15 (1.5)	21	0	2 (0.2)
Erythrodermic atopic dermatitis	0	0	0	0	0	0	0	1 (50.0)	0	0	0	1	0	0
Exfoliative rash	0	3 (2.1)	10 (7.1)	6 (4.3)	1 (0.7)	2 (1.4)	52 (37.1)	45 (32.1)	2 (1.4)	13 (9.3)	6 (4.3)	0	0	0
Generalised bullous fixed drug eruption	0	0	2 (6.3)	0	0	0	12 (37.5)	1 (3.1)	3 (9.4)	8 (25.0)	0	6 (18.8)	0	0
Oculomucocutaneous syndrome	0	3 (16.7)	0	0	0	0	6 (33.3)	5 (27.8)	1 (5.6)	1 (5.6)	2 (11.1)	0	0	0
Severe cutaneous adverse reaction	0	1 (2.4)	2 (4.8)	3 (7.1)	0	0	2 (4.8)	23 (54.8)	4 (9.5)	6 (14.3)	0	1 (2.4)	0	0
SJS-TEN overlap	2 (3.9)	0	1 (1.9)	0	0	1 (1.9)	15 (28.9)	14 (26.9)	0	19 (36.6)	0	0	0	0
Skin necrosis	29 (8.5)	52 (15.3)	25 (7.4)	8 (2.4)	0	14 (4.1)	20 (5.9)	162 (47.7)	10 (2.9)	16 (4.7)	1 (0.3)	3 (0.8)	0	0
Stevens-Johnson syndrome	46 (4.1)	36 (3.2)	42 (3.7)	14 (1.2)	3 (0.3)	14 (1.2)	280 (24.8)	388 (34.4)	74 (6.6)	193 (17.1)	10 (0.9)	23 (2.0)	1 (0.09)	3 (0.3)
Target skin lesion	0	1 (5.0)	0	2	0	1 (5.0)	10 (50.0)	1 (5.0)	2 (10.0)	1 (5.0)	2 (10.0)	0	0	0
Toxic epidermal necrolysis	79 (4.9)	72 (4.5)	95 (5.9)	7 (0.4)	8 (0.5)	35 (2.2)	441 (27.5)	401 (25.0)	116 (7.2)	290 (18.1)	14 (0.9)	34 (2.1)	6 (0.4)	8 (0.5)

Table S6. Top-10 drugs with the highest reporting frequencies.

SMQ SCAR	DRESS	TSE	TEN	EM	SJS	DB	AGEP	DEG	CV	SN
Dupilumab<br>244 (2.14%)	Piperacillin and beta-lactamase inhibitor<br>115 (4.32%)	Amoxicillin and beta-lactamase inhibitor<br>44 (2.66%)	Pembrolizumab<br>48 (2.99%)	Dupilumab<br>58 (5.81%)	Pembrolizumab<br>51 (4.53%)	Linagliptin<br>206 (23.09%)	Clindamycin<br>46 (5.08%)	Dupilumab<br>85 (19.77%)	Apixaban<br>41 (7.47%)	Empagliflozin<br>15 (4.41%)
Piperacillin and beta-lactamase inhibitor<br>227 (2.0%)	Levofloxacin<br>89 (3.34%)	Clindamycin<br>42 (2.54%)	Lamotrigine<br>46 (2.86%)	Pembrolizumab<br>43 (4.31%)	Enfortumab vedotin<br>40 (3.55%)	Pembrolizumab<br>19 (2.13%)	Piperacillin and beta-lactamase inhibitor<br>39 (4.31	Clindamycin<br>Apalutamide<br>9 (2.09%)*	Dupilumab<br>16 (2.91%)	Enoxaparin<br>13 (3.82%)
Pembrolizumab<br>225 (1.98%)	Vancomycin<br>76 (2.85%)	Ceftriaxone<br>41 (2.48%)	Enfortumab vedotin<br>44 (2.74%)	Nivolumab<br>36 (3.61%)	Lamotrigine<br>32 (2.84%)	Acetylsalicylic acid<br>13 (1.46%)	Enoxaparin<br>32 (3.54%)	Ixekizumab<br>Upadacitinib<br>8 (1.86%)*	Amiodarone<br>14 (2.55%)	Abatacept<br>12 (3.53%)
Levofloxacin<br>222 (1.95%)	Carbamazepine<br>70 (2.63%)	Efavirenz<br>40 (2.42%)	Paracetamol<br>38 (2.37%)	Lenvatinib<br>31 (3.11%)	Levofloxacin<br>26 (2.31%)	Fluconazole<br>Enfortumab vedotin<br>12 (1.35%)*	Amoxicillin and beta-lactamase inhibitor<br>23 (2.54%)	Acetylsalicylic acid<br>Pemetrexed<br>7 (1.63%)*	Clindamycin<br>Levofloxacin<br>13 (2.37%)*	Apixaban<br>11 (3.24%)
Linagliptin<br>220 (1.93%)	Clindamycin<br>Meropenem<br>Levetiracetam<br>54 (2.03%)*	Pembrolizumab<br>Carbamazepine<br>31 (1.87%)*	Levetiracetam<br>33 (2.05%)	Ipilimumab<br>24 (2.40%)	Ibuprofen<br>25 (2.22%)	Ibuprofen<br>Levofloxacin<br>11 (1.23%)*	Terbinafine<br>22 (2.43%)	Metronidazole<br>Diclofenac<br>Nivolumab<br>Mometasone<br>5 (1.16%)*	Nivolumab<br>11 (2.00%)	Rituximab<br>9 (2.65%)
Clindamycin<br>203 (1.78%)	Metronidazole<br>Allopurinol<br>46 (1.73%)*	Levofloxacin<br>26 (1.57%)	Ibuprofen<br>31 (1.93%)	Pneumococcus vaccine. purified polysaccharides antigen conjugated<br>Diphtheria-haemophilus influenzae b-pertussis-poliomyelitis-tetanus vaccine<br>23 (2.30%)*	Nivolumab<br>24 (2.13%)	Ceftriaxone<br>10 (1.12%)	Hydroxychloroquine<br>21 (2.32%)	Other drugs**	Etanercept<br>10 (1.82%)	Tocilizumab<br>7 (2.06%)
Carbamazepine<br>183 (1.61%)	Sulfamethoxazole+trimethoprim<br>45 (1.69%)	Lamotrigine<br>25 (1.51%)	Carbamazepine<br>Meropenem<br>29 (1.81%)*	Carbamazepine<br>Cefoperazone + sulbactam	Carbamazepine<br>Levetiracetam<br>20 (1.77%)*	Nivolumab<br>Carboplatin<br>9 (1.01%)*	Amoxicillin<br>18 (1.99%)		Secukinumab<br>9 (1.64%)	Dupilumab<br>Rivaroxaban<br>6 (1.76%)*

				19 (1.90%)*						
Ceftriaxone<br>173 (1.52%)	Enoxaparin<br>44 (1.65%)	Nivolumab<br>23 (1.39%)	Ceftriaxone<br>27 (1.68%)	Carboplatin<br>Bevacizumab<br>Celecoxib<br>18 (1.80%)*	Bevacizumab<br>19 (1.69%)	Hydroxychloroquine<br>Valproic acid<br>Metronidazole<br>Rivaroxaban<br>Paracetamol<br>Iron. parenteral preparations<br>8 (0.90%)*	Ceftriaxone<br>17 (1.88%)		Amoxicillin and beta-lactamase inhibitor<br>Rituximab<br>Rivaroxaban<br>Ipilimumab<br>Atorvastatin<br>Tocilizumab<br>Ibrutinib<br>7 (1.28%)*	Nivolumab<br>Valproic acid<br>Gemcitabine<br>Cyclophosphamide<br>5 (1.47%)*
Nivolumab<br>160 (1.41%)	Ceftriaxone<br>43 (1.61%)	Piperacillin and beta-lactamase inhibitor<br>Amoxicillin<br>Enoxaparin<br>22 (1.33%)*	Piperacillin and beta-lactamase inhibitor<br>24 (1.49%)	Paclitaxel<br>Atezolizumab<br>17 (1.70%)*	Ipilimumab<br>Sulfamethoxazole + trimethoprim<br>18 (1.60%)*	Dupilumab<br>Apixaban<br>Pantoprazole<br>Regorafenib<br>Empagliflozin<br>7 (0.78%)*	Levofloxacin<br>14 (1.55%)		Acetylsalicylic acid<br>Methotrexate<br>Rosuvastatin<br>6 (1.09%)*	Methotrexate<br>Pembrolizumab<br>Bevacizumab<br>Prednisone<br>Dabigatran etexilate<br>Mycophenolic acid<br>Interferon beta-1b<br>Glatiramer acetate<br>4 (1.18%)*
Amoxicillin + beta-lactamase inhibitor<br>147 (1.29%)	Rifampicin<br>39 (1.46%)	Acetylsalicylic acid<br>Ciprofloxacin<br>19 (1.15%)*	Levofloxacin<br>Acetylsalicylic acid<br>23 (1.43%)*	Acetylsalicylic acid<br>16 (1.60%)	Piperacillin + beta-lactamase inhibitor<br>17 (1.51%)	Levetiracetam<br>Rosuvastatin<br>Furosemide<br>Metformin<br>6 (0.67%)*	Ibuprofen<br>Pantoprazole<br>Dexamethasone<br>12 (1.33%)*		Ixekizumab<br>Ceftriaxone<br>Metformin<br>Methylprednisolone<br>Infliximab<br>Tocilizumab<br>3 (0.88%)	Acetylsalicylic acid<br>Methylprednisolone<br>Tocilizumab<br>Furosemide<br>Lenalidomide<br>Carboplatin<br>Dexamethasone<br>Prednisolone<br>Paclitaxel<br>Warfarin<br>Denosumab<br>Leflunomide<br>Vincristine<br>Doxorubicin<br>Epirubicin<br>Nadroparin<br>Lauromacrogol 400<br>3 (0.88%)*

*Same frequencies for all listed drugs
**Drugs with n<5.

Table S7. Data for PRR and ROR calculations.

Drugs	A	B	C	D
Dupilumab	244	26244	6767	874743
Piperacillin and beta-lactamase inhibitor	227	2781	6784	898206
Pembrolizumab	225	17414	6786	883573
Levofloxacin	222	6895	6789	894092

Linagliptin	220	1690	6791	899297
Clindamycin	203	1968	6808	899019
Carbamazepine	183	2625	6828	898362
Ceftriaxone	173	10809	6838	890178
Nivolumab	160	11152	6851	889835
Amoxicillin and beta-lactamase inhibitor	147	4161	6864	896826
